# Book Reviews

**Published:** 1804-09-01

**Authors:** 


### CRITICAL ANALYSIS

OF THE

### RECENT PUBLICATIONS

ON THE

### DIFFERENT BRANCHES OF PHYSIC, SURGERY, AND MEDICAL PHILOSOPHY.

---

*Medical Report of Cases of Inoculation and Re-inoculation with Variolous and Vaccine Matter: with some Cases of casual Exposure to Small-Pox Contagion, subsequent to Vaccination. By* JOHN ROLLO, M. D. &c. &c. 8vo. pp. 23. London, 1804.

It appears, by this short Report, that Mr. Goldson's pamphlet had produced no small degree of doubt respecting the permanent efficacy of vaccination even in the mind of Dr. Rollo. We conclude, therefore, that it has produced more than doubt in many minds far less enlightened, and less capable of judging of medical evidence than he is. We rejoice, however, for the occasion which threw a subject, so interesting to the public, into hands so well qualified for its investigation. Dr. R. appears to have availed himself of every circumstance which could render his experiments decisive of the question at issue. The season of the year, the activity of the

 small-pox small-pox matter employed, attention to its effectual insertion into the arms, and, lastly, by inviting a considerable number of respectable practitioners to witness the progress of the cases. The result of these experiments, and many others, instituted with the same view, in various parts of the kingdom, we conclude, has set the matter at rest for some time at least.

"So far as our cases go," says Dr. R. "we could hardly have refrained from drawing individual inferences; but this, and any other explanation necessarily arising from them, we at present decline. Except in observing, that, from the whole of this report, the subsequent remarks so strongly arise, that we think it proper to subjoin them: namely, that the vaccine inoculation resists the action of the variolous disease; that when this has been *apparently* diminished as a preventive power, the variolous disease has been much milder, and less compleat; but how far time may farther influence the vaccine power, time only can decide; though at present it remains entirely in favour of vaccination, as deducible from our report."

"It is with the greatest satisfaction to us, that it will be perceived, that of the five hundred and fifty we have vaccinated, not more than thirteen have been in any degree affected by re-inoculation, or casual exposure to variolous matter; and in these, any disease induced has been extremely mild, and some doubt may arise whether it was really variolous constitutional affection or not. It is also to be recollected, many of the remaining five hundred and thirty-seven cases of cow-pock have been occasionally exposed to variolous influence without effect. Therefore, so far as our experience extends in vaccination, it is completely supported. From every analogy, it is reasonable to suppose that time will establish the demonstration of its full and permanent power."

---

*Minutes of some Experiments to ascertain the permanent Security of Vaccination, against Exposure to the Small-Pox. To which are prefixed, some Remarks on Mr. Goldson's Pamphlet. With an Appendix, containing Testimonials and other Communications from many of the most respectable Medical Men in this Neighbourhood.* By RICHARD DUNNING, *Surgeon, and Secretary to the Dock Jennerian Institution.* 8vo. pp. 120. Plymouth.

THE enquiry excited among Medical Men throughout the British Empire by Mr. Goldson's pamphlet, seems likely to obtain for them, in the world at large, a character for vigilant observation on the important subject which does such honour to our nation. The discussions it has provoked, and the train of experiments it has induced, will be every where attentively regarded; and when the alarms which its extensive circulation has occasioned shall have subsided, the Jennerian inoculation must be universally received with increased confidence. Like our native oak, the flourishing plant plant (vacciola) shall become but more firmly rooted by the blasts which may have threatened to overturn it.

Mr. Dunning says, very liberally, of the work which, at least, has given rise to very valuable observations, "Mr. Goldson's pamphlet, I fear, is much too well written not to excite a very general interest; and I lament to add, not to occasion a vast deal of misery and distraction in many thousands of families; at the same time I am most ready to admit, and I admit it with great satisfaction, that his observations, &c. are written apparently with too much candour for me to doubt a moment his willingness fully to retract them, whenever he shall see occasion to do so. Indeed, the whole *Tenor* of the pamphlet carries conviction to my mind, that the author is not a bigot, who, if wrong, will not be convinced."

Mr. Dunning, as soon as he had read Mr. Goldson's pamphlet, availed himself of the earliest opportunity to set on foot the tests of variolation and exposure to the fullest infection of the small-pox that could be devised; in which he had the concurrence of Doctors Remmet, Woolcombe, and May; Messrs. Little, Fuge, Smith, Lower, Penkivel, Bone, Veitch, and Sargent. Many of his little patients, whom he had vaccinated at different periods in the course of the last four years, had been constantly breathing for many weeks a highly variolated atmosphere. He had vaccinated more than a thousand subjects since the latter end of 1799; and as he had never met with a single instance of subsequent small-pox, though many of them had been subjected to variolation, and many constantly and fully exposed to casual infection, nor known a case in the practice of any surgeon in his town and neighbourhood, he was naturally convinced of its permanent efficacy.

We pass over upwards of thirty pages, as being principally theoretical, and in some parts perfectly hypothetical, in order to come to the facts, in which all the world is concerned.

Mr. Dunning, a fond parent, had his child, ten months old, whom he had vaccinated eight months before, inoculated (January 23, 1802) from a woman ill of the most malignant small-pox, of which she died three days afterwards. "An early and considerable local affection on the arm took place, and gradually progressed till the seventh or eighth day, when it began to shade, and in a few days dried off. This inoculation was neither attended with the slightest constitutional effects, nor followed by the smallest vestige of the small-pox. This child he also inoculated again for the small-pox in July 1804; and, on this occasion, as well as the former, had the child taken into the close room of the sick, thus exposing it to the contagion of the disease as well as the infection by the lancet.

At the time of the latter inoculation Mr. Dunning, had another son also submitted to the operation. "The eldest boy is twelve years old, and had been inoculated more than eleven years before, and suffered a good deal from the small-pox; the susceptibility of small-pox small-pox in our family is very remarkable." The effect of the variolous inoculation was nearly the same on the younger child as it had been two years and a half before; on the elder, a slower but rather greater effect of the same kind was produced.

"The benign cow-pock," says the author, has now, in the person of my youngest child, completely and unequivocally resisted, *at different and distant periods*, the poison of small-pox." Almost immediately on the effect of the inoculation on this child being ascertained, it was discovered that children were dying of scarlatina within a few yards of the house, when Mr. Dunning in a few hours removed his family from the village; but the infant was already infected; and on this he observes, "No circumstance can more strongly mark the susceptibility of contagion in this child than his taking on so readily the scarlatina, the previous inoculation having gone through all its stages three days before the seizure: this circumstance places, if possible, in a more striking point of view, the unsusceptible state of the child's system, with respect to the poison of the small-pox; and indeed, although it has been an afflicting circumstance to the child and my family) gives an additional weight to the experiments which I hope and believe the utmost ingenuity of any man, the most adverse in the world to the cow-pock, cannot lessen."

In addition to the evidence contained in Mr. D's Minutes, he has given a valuable body of correspondence and extracts, all tending to establish the same satisfactory conclusion as the Minutes had done. We here also find occurrences and cautions similar to those lately laid before the public by Mr. Ring.

---

*Surgical Observations, containing a Classification of Tumours, with Cases to illustrate the History of each Species; an Account of Diseases which strikingly resemble the Venereal Disease, and various Cases illustrative of different Surgical Subjects. By* John Abernethy, F. R. S. &c. &c. 8vo. pp. 263. 1804.

[ Continued from pp. 176—179. ]

After this, the author slightly mentions another *genus* of tumours, which he calls osseous, and illustrates with a case. The division concludes with the following observations:

"Vascular tumours also may doubtless become converted into a substance resembling cartilage, like those found in joints; and their hardness might then exclude them from the genus sarcoma. I have not however met with such instances, though it is not very uncommon to find a substance resembling cartilage intermixed with the other vascular substance of a sarcocele of the testis.

"The diseases which I have been describing may be considered as edifices which are built up by diseased actions, and in which those diseased actions continue to reside. The actions themselves do not admit of examination, though the structures do which they erect erect. Therefore, as Dr. Baillie has observed, it is by an examination of diseased structure that we must be slowly led to a knowledge of diseased actions. It does not follow as a certain consequence, that similar diseased actions will, in every instance, produce precisely the same diseased structure; though it is highly probable that they will do so in general. This observation would diminish our surprize if, in some rare instances, we found cancer existing where a cancerous structure was not strikingly manifest; or if, in others, a structure like that of cancer, was observed where no cancerous actions were apparent. The scirrhus tumours, which form beneath the peritoneal covering or lining of the uterus, have something of the structure of cancer, and yet they are not cancerous. In all cases where tumours are formed we must suppose an increase, and, in some degree, a disordered action of the vessels which form them, but, in many these actions possess but little diseased peculiarity. As in every case of growth, in the re-production of destroyed parts, the gelatinous substance of the blood is first deposited, and afterwards rendered vascular, therefore I have considered a tumour formed in this manner as one of the most simple kind, and possessing the least of diseased peculiarity; but I am aware that I may have included under this general character tumours of essential different natures. In the adipose sarcoma there must be some peculiarity in the arrangement and actions of vessels which form this tumour; but it must be accounted a natural rather than a morbid peculiarity. The pancreatic sarcoma, I should suppose, differed but little from the first species. It may be considered as a new growth characterized merely by the peculiarity of its appearance, in consequence of its being separated into many distinct parts, which sometimes cohere by a looser kind of texture, and sometimes are separated by a firmer substance. The connecting medium appears like the thickened cellular substance of the part in which the newly organized matter is formed. Indeed, I have sometimes pressed out the separated portions of this substance from the connecting medium which environed them. In the mammary sarcoma I suspect some diseased peculiarity to exist, as has been mentioned in speaking of that subject. In the tuberculated sarcoma the predisposition to that disease seems general on the part of the constitution. In the medullary sarcoma the disease seems local, in the first instance, and propagated by means of the absorbing vessels to their glands, and frequently in a course retrograde to that which the absorbed fluids would naturally take; but in the advanced state of the disease the morbid disposition appears to be general. In carcinomatous sarcoma the disease appears to begin in a point or small district, and to extend in every direction, as rays do from a centre, affecting every surrounding part whatever may be its nature. The diseased actions also, though they may be at times more violent or more tranquil, never cease. This disease is also extended through the medium of the absorbing vessels in the direction which the absorbed matter would naturally take."

Our

Our readers will lament with us that this language, how well so ever the facts may be supported, is much too figurative for practical purposes. But, lest we should be accused of partiality, we again refer the reader to the work itself.

The next section is on diseases resembling syphilis. This is introduced with the following well deserved tribute to the memory of Mr. Hunter.

"Having thus ventured again to appear before the public, I shall take the opportunity of exciting its attention to some cases which have occurred to me of diseases resembling syphilis. Mr. Hunter, in his excellent Treatise on the Venereal Disease, has related several cases supposed to be of that nature, and some of which were certainly not so, as they got well without mercury; but in the greater number the employment of this medicine rendered their nature doubtful. Mr. Hunter also, who was as cautious in drawing conclusions as he was accurate in making observations, expresses himself in many instances so diffidently on the subject, as, in my opinion, not sufficiently to impress the minds of his readers with the certainty, importance, and frequency of such facts. He concludes his observations by intimating, 'that undescribed diseases, resembling venereal, are very numerous, and that what he has said is rather to be considered as hints for others to prosecute this inquiry further, than as a complete account of the subject.' As it has occurred to me very frequently to meet with such cases, and as the necessity for discriminating them from venereal diseases appears to me of the highest importance, I shall prosecute the subject by relating some unequivocal cases of diseases strikingly resembling syphilis, and which, however, were not so, provided it be admitted that syphilis does not spontaneously get well without the aid of medicine."

This division of the work is principally made up of very instructive and important cases. It would be unjust to make any observations on the want of accurate discriminations, or that no arrangement is attempted. The author's object is evidently to impress on his readers a closer attention to that variety of sores which appear on the genitals, and of ulcers, spots or nodes, which attack other parts of the body, and are too indiscriminately confused with venereal complaints. In doing this, he has very judiciously selected some striking cases, which cannot fail to impress the young practitioner in a lively manner; to such we recommend the careful and repeated perusal of the whole; we shall only extract the following passage, as it does credit to the author's accuracy and candour.

"With respect to sores that are not venereal, the difficulties of investigation are greatly multiplied. If a description cannot be given of venereal sores, it seems almost absurd to say any thing of those multiform sores produced by infectious matter, the qualities of which may be probably variously modified, and the effects of which appear equally liable to modification from peculiarities of constitution.

Yet in this intricate subject there are certain facts which can be distinctly observed, and deserve attention. Some of these sores spread by ulceration, and some by sloughing, of which instances are related in the first section of this paper. Even Celsus has described several species of sores which, as Dr. Adams has observed, we are acquainted with in the present day. I have never seen that phagedænic ulcer, which suddenly sloughs, affect the constitution; neither do I believe that surgeons in general have remarked it; those who regard all these sores as venereal, attribute the absence of secondary symptoms to the chancre having been removed by the sloughing of the surrounding parts. Yet in the case related by Mr. French in Mr. Hunter's Treatise on the Venereal Disease, secondary symptoms did occur from a sore of this kind, and got well without mercury. It may therefore, perhaps, be doubted whether this disease be not an aggravated form of the sore which sloughs more slowly, and from which the constitution is much more frequently affected. As I consider any observations that I have made on these sores to be incomplete and therefore not to be depended upon, and Dr. Adams having restricted the term Phagedæna to one kind of destructive sore, I feel more inclined to leave it as a generic term for all these sores, and to divide them into species according to their peculiar characters. Then we may describe them as ulcerating phagedænic sores, and sores which spread by sloughing. Again, the ulcerating or sloughing process may extend not in all but in particular directions, and the sloughs may take place from the edges or from the whole surface. As Dr. Adams has treated these subjects at large, I refer the reader to his book; but I will take upon me to describe one species of sore which frequently occurs, and is generally treated as venereal, but which I am convinced is not so.

"The sores alluded to generally break out in succession, and sometimes after a considerable interval of time; which circumstance, if remarked, would render it improbable that they arose from infection of the ulcerated part, since such sores would probably be contemporary. The ulcer is at first inflamed, and spreads ordinarily to the size of the finger nail: its circumference is thickened; it throws out new flesh, which rises above the surrounding skin; sometimes there is an appearance of several little cells or spaces in the interstices of the granulations, if they may be called so, owing to the whole ulcer not producing new flesh in an equal degree. These sores are slow in healing under any mode of treatment, and they generally get well in the same succession as they broke out. They sometimes form in a circle round the orifice of the prepuce, and cause a contraction in that part after they have healed. I do not mean to say that all sores occupying this situation are not venereal, but merely to state that sometimes after a gonorrhœa of the prepuce, either originally occurring there, or having happened by a metastasis of disease from the urethra, sores do break out in this situation at a remote period from the receipt of the infection, fection, which are not venereal. They seem to be the consequence of an irritated state of the prepuce, from which there is sometimes a slight general discharge, like that which takes place when the gonorrhœa shifts its situation from the mouth of the urethra, and becomes the gonnorrhœa of the prepuce. The glands in the groin sometimes swell from irritation in these cases, and generally subside again, though I have known them suppurate; but I never saw any secondary symptoms succeed to this species of ulcer.

"In the earlier part of my practice, in conformity to general rules, I used to give mercury in these ulcers to secure the constitution against infection, whilst I tried to heal the sores as speedily as I could by topical applications. Slightly destroying the surface with the argentum nitratum every second day, and dressing with the solution of zincum vitriolatum, were the local means which seemed to be most successful. An attention to the history of the disease, and frequent applications for advice from persons who had been severely and unavailingly salivated for the cure of this species of sore, soon emboldened me to abstain from the use of mercury, and I have never found, though I have met with a considerable number of instances, that I have in this respect acted wrong."

The last division of the work consists of *various cases illustrative of different surgical subjects.* It commences with injuries to the head, in which our author again instances several cases of fracture, for which the application of the trephine was unnecessary, and others in which it could not be attended with any advantage. They are related with his usual accuracy, and the inferences drawn are such as we might expect from Mr. Abernethy. The last case, though not strictly in point, is peculiarly important, for which reason we shall transcribe the whole.

"A man was gored in the neck by a cow. The horn entered by the left side of the cricoid cartilage, and penetrated as far as the vertebræ; it then passed upwards on the bodies of those bones, nearly as high as the bottom of the skull, afterwards it came out behind the angle of the jaw, exposing and in some degree injuring the parotid gland in its passage, and lacerating the skin of the face as high as the middle of the ear. In its course it had passed beneath, and torn the internal carotid artery, and all the primary branches in front of the external carotid artery. The former vessel was not however entirely rent asunder, so that the general course of the artery and its connection with the cranium remained in the usual state. Notwithstanding the size of the vessels which had been torn, they did not immediately bleed; the wound was therefore closed and bound up. The blood was soon observed to flow in streams down the neck, nor could any general pressure upon the wound prevent hæmorrhage. In this state the man was conveyed to St. Bartholomew's hospital, but he lost a large quantity of blood before his arrival.

"The patient was laid upon a bed, and before the wound was opened, one of the students firmly compressed the trunk of the carotid carotid artery against the lower cervical vertebræ. We found upon the first inspection of the wound, that this pressure prevented any hæmorrhage; yet upon the occasional motions of the patient, and upon accidental variations in the pressure made on the vessel, the blood gushed from the bottom of the wound so suddenly and in such quantities as to prevent any accurate examination. The man was very unquiet; he complained much of the pressure, and was greatly distressed by a sensation of suffocation, which compelled him constantly to attempt to expectorate. Under these circumstances our first endeavours were to tie the more superficial arteries; but the edges of the wound being lacerated, the first ligatures which we endeavoured to make, tore away portions of the flesh, and did not secure the vessels.

"The situation of the patient became every moment more desperate, he really seemed choking, his extremities became cold, and his pulse was scarcely to be felt: his struggles also, which could not be controlled, made the pressure on the trunk of the artery very precarious. It was deemed necessary to enlarge the wound to get at the trunk of the carotid artery, and an incision was made between that vessel and the trachea, in a direction parallel to each of these parts. I had now the power of passing my finger beneath the trunk of the carotid artery; and of effectually compressing it between that finger, and my thumb which was placed opposite to it, upon the integuments of the neck.

"I had now leisure to examine the wound with my other hand, and felt that the pharynx had been separated from the vertebræ of the neck, and had fallen against the larynx: its irritation on that organ was probably the cause of the sensation of suffocation which the patient suffered. There did not appear any reason to believe that the pharynx was wounded; for though the patient was constantly spitting, the mucus was not mixed with blood. Finding that the moment I remitted the pressure on the carotid, the blood gushed out from so many orifices, and in such a torrent from the bottom of the wound, I resolved to pass a ligature round the trunk of the carotid at the part where I had been compressing it, and which was about an inch below its division. This ligature I thought might be made to serve as the tourniquet in amputation, for I could with it compress the artery so as to prevent the wounded parts becoming obscured by blood, and by slackening it I might gain information with regard to the situation of the ruptured vessels.

"Should it become necessary at any time to tie the carotid artery, I am convinced that it may be done without much difficulty or danger, even without an accurate dissection of the part. If the incision be made on that side of the artery which is next the trachea, where no important parts can be injured, as was done in the present instance, the finger can then be passed behind the artery so as to compress it. The vessel being sufficiently bulky and firm, to make its form and outline distinctly perceptible, a needle may then be passed behind the artery, as near as possible to that edge of it it which is next to the internal jugular vein; there can be little risk of wounding that vessel, or of including in the ligature the eighth pair of nerves which lies between them. In attempting to secure the carotid artery, I passed behind it in the manner described, a blunt hook with an eye in the point, and having previously introduced a ligature into it, I drew back the instrument and thus enclosed the artery.

"When I compressed the vessel by tightening the knot of the ligature, I did it slowly, and with a watchful attention to the sufferings of the patient; for I cannot but suppose that had the nerve of the eighth pair been included, his complaints would have sufficiently denoted that circumstance. But the compression of the ligature did not seem to make the least difference in the general state of the patient, whilst it completely prevented the further effusion of blood. With a knife and dissecting forceps I then exposed the lacerated vessels, and found that the primary branches of the external carotid artery had been torn off from the trunk. By drawing upwards the ligature which encircled the trunk of the artery, I made the internal carotid tense, so that its course and ruptured state could be distinctly felt. The ligature on the trunk was slackened, and the gush of blood further confirmed the laceration of the internal carotid artery. I had now the alternative of securing the ligature, which I had already made on the trunk of the vessel, or of tying the branches separately. I preferred the former, and it should be observed that the man had now lain ten minutes or more, without any blood being carried to the brain by the left carotid; and during that period he had recovered from his extreme faintness, appeared perfectly sensible, and as well as could be expected in a person, considering that he had lost so large a quantity of blood. The ligature being now made secure, the wound was brought together by stripes of plaster; and in this state warm milk was given to the patient to drink, in order to learn what would be the effect of his efforts to swallow, and to ascertain, as far as possible, whether there was any wound in the pharynx or œsophagus. The patient swallowed about a quarter of a pint of this fluid with difficulty, and with the frequent excitement of coughing. No milk however came through the wound, and I concluded that all the difficulty of deglutition arose from the unnatural state in which the muscles of the pharynx were placed, in consequence of their detachment from the vertebræ These circumstances happened between four and five o'clock in the afternoon, and when I saw the patient again between nine and ten, his state seemed greatly amended. He had several times taken warm milk, and the difficulty of deglutition had abated. His pulse was now moderately full and strong, and not very frequent. It therefore appeared that the apparently dying state of the man, which at one time had alarmed us, proceeded rather from the sudden discharge of blood, than from the quantity, however considerable, which had been lost. The patient also appeared tranquil, and perfectly rational, and though prevented prevented from speaking much, he expressed himself satisfied in his situation.

"On the whole I was led to form a favourable expectation of the progress of the case, as far as related to the effects which a ligature on one carotid would have on the economy of the brain. I was next morning mortified to learn that the patient had been unquiet, and feverish during the night, that he had become delirious, that he had been several times affected by slight convulsions, which had increased; and that when liquids were now given to him, they passed through the wound, and he could scarcely swallow any thing. The pulse of the patient was now about 130 in a minute, and hard, and his skin was hot. He lay inattentive to external objects, but probably not insensible, for the pupils of his eyes were contracted; and when the lids were opened, in order to examine them, he shut them quickly, and as it were, impatiently. It had been remarked, that the left side of the body was more convulsed than the right.

"As we had it not in our power easily to give medicine, I introduced a small hollow bougie through the right nostril into the œsophagus, and immediately injected half a pint of milk and water, and sixty drops of tincture of opium; that I might learn the effects of that medicine under the present circumstances. The patient shortly after broke out into a most profuse sweat, and the convulsions were quieted by the opium. The convulsions when thus mitigated by opium, might be described as violent tremors of the left side of his body, but the right side remained motionless; to which curious fact I particularly attended. I placed his right arm across his breast, from which situation it did not afterwards stir. I could not, however, perceive any distortion of the face to the opposite side, and the pupils of both eyes were equally contracted. When I saw the sweat break out on the taking of opium, and the nervous irritation diminished by its operation, I was then more forcibly struck than I had been before with the similarity of this patient's situation to that of a person suffering from the effects of concussion of the brain, some time after the accident, when the inflammation often succeeding to it, had begun to take place.

"I even questioned if it might not be right to take blood from the temporal artery, which was seen beating violently. I thought however the general opinion would be against such practice, and I only applied a blister to the head. Twenty drops of tincture of opium were directed to be given to the patient every third or fourth hour, with a view to mitigate the convulsions, which it appeared to do. Milk and water was also occasionally given, in proportion to the degree of perspiration. No remarkable change of symptoms took place, but the strength of the pulse gradually declined, and at ten o'clock at night he had a severe convulsion fit, and immediately after died. His death happened about thirty hours after the ligature was made on the carotid artery.

"The body was examined on the following day. The brain appeared to have suffered a considerable degree of inflammation.

The vessels of the pia mater appeared as if they were injected, and in many places upon the surface of the convolutions of the cerebrum, there even seemed an effusion of blood producing that appearance usually termed bloodshot. There was a very considerable deposition of gelatinous substance between the tunica arachnoidea, and the pia mater. The vessels passing through the substance of the brain, though fuller than common, were not particularly turgid. A considerable quantity of water, of a light brown colour, and slightly turbid appearance, was found in the ventricles, whilst the firmness of the sides of those cavities sufficiently indicated that the collection had not preceded the accident. On examining the neck, the carotid artery was found to be the only part included in the ligature. The superior thyroideal, lingual, and facial branches of the external carotid, were torn off from the trunk, and the internal carotid was rent across, as has been already mentioned.

" Neither the trunk of the eighth pair of nerves, nor the great sympathetic, nor those of the tongue, appeared to have suffered injury. The superior laryngeal and the descending branch of the ninth pair were the chief nerves injured by the accident. These circumstances are mentioned, to enable the reader to form his own judgment on the probability of the symptoms which occurred being produced by nervous injury or irritation.

" That the disorder and death of this man is not to be attributed to the quantity of blood which he had lost, appears clearly to me, not only from the degree of plenitude and power of the vascular system which remained, but because I had seen many patients in the hospitals, who had divided most of the primary branches of the external carotid artery in the attempt at suicide: and who, after surviving a few days, perished in consequence of the loss of blood which they had sustained, but with a train of symptoms very different from those which occurred in the present instance.

" Some persons may perhaps be inclined to attribute inflammations of the brain to nervous injury or irritation. I have taken notice of all the injury discoverable by dissection, and have further to observe, that we frequently see larger nerves lacerated in wounds without the production of such symptoms; and the tranquil state of the patient till the inflammation of the brain came on, opposes such an idea. Upon reflection, I can form no other opinion of the case than that which first struck me, which is, that though the stopping the supply of blood to the brain did not for several hours produce any apparent derangement in the functions of that organ, yet such a state was gradually occasioned by it, and which was attended like the effects of concussion of the brain, with inflammation. It further appeared, that when the combined effects resulting from the derangement, and the inflammation, were manifested together, the state of the patient much resembled that of a person who had suffered concussion.

" The different states of the two sides of the body ought not I think to pass without further notice. Although the right side could could not be positively said to be paralytic, yet in my opinion it approached to that state.

"It has been already observed, that a double construction might be put upon the symptoms, yet as the inflammation of the brain was equal on both sides, we might naturally expect the whole body to suffer equally. Should the state of the right side have been, as appears most probable, an approach to a state of paralysis, it would surely be considered as peculiarly curious. An effusion of blood in the left hemisphere of the brain would affect the opposite side of the body in the same manner, that cutting off the supply of blood to the left side appears in this instance to have done. I forbear to speculate on this subject; the fact which I have mentioned seems to deserve notice, and though at present it must stand alone, it may in future receive addition, and, when thus supported, be applied to some useful purpose in physiology.

"I have thought it right to record this case, not merely because it is curious, but because it affords some useful practical hints, as to the conduct to be pursued when a person has divided the large primary branches of the carotid artery in an attempt at suicide. It may be allowable also to mention, in relation to this latter subject, the great advantages which appear to me to arise from the immediate introduction of a small elastic catheter, passed through the right nostril, down the œsophagus, nearly as far as the stomach, in the manner practised by Dessault, in the cure of a person wounded by a pistol ball.

"A patient in such a state is not under the necessity of frequently swallowing nourishment, which act tears open the wounded parts, and causes inflammations in them, and produces such a secretion of mucus as excites almost constant cough, increasing the disturbance of the wounded parts.

"The introduction of a small elastic catheter may be easily accompolished in the first instance, though not without difficulty, after the sensibility of the parts have been increased by inflammation; and from the benefit I have seen derived from it, I should not hesitate to do it in all cases of extensive wounds of the throat, where the larynx or trachea is divided, even though the pharynx and œsophagus may be uninjured. It seems to me also that a similar plan of conduct is very suitable to strictures of the œsophagus."

Two cases of aneurism of the femoral artery are next related, in both which the operation proved fatal, or rather the patient died after the operation. However, as the case is circumstantially related, we shall leave the reader to form his own conclusions. In the second case, the death of the patient (a lady) is imputed to a peculiar irritability of constitution, which produced so general a disturbance that the part sloughed, in consequence of which hæmorrhage ensued, which in the end proved fatal. Mr. Abernethy introduces these cases by assuring us, that since his last publication he has in other instances found the operation for the aneurism there recommended uniformly successful.

Some observations follow on the operation of puncturing the urinary bladder. Here the author recommends the puncture above the os pubis instead of by the rectum. We confess ourselves not prepared to maintain all the objections which must occur to every practitioner, against this operation; we therefore leave it to abler hands, whose objections, we doubt not, will appear before us.

A case of tic doloreux follows, which gives rise to some useful reflections on the disease, and on the physiology of the brain and nerves. Lastly, we have some valuable practical remarks on the removal of loose substances from the knee-joint.

Thus have we gone through this miscellaneous performance; and from our account the reader will expect much information from the perusal of the whole. In this, he will not be disappointed. But while we thus recommend the book to our readers, we cannot refrain from a few words of advice to the author.

The multifarious materials of such a work makes us less surprized, if all should not be as complete as we expect from a surgeon of our author's rank. In answer to this we may be told, that want of leisure has prevented that accuracy which men less engaged in practice may attain. But when men of less notoriety than Mr. Abernethy are inaccurate, the consequence to the public is much less in proportion as their authority is less. We would not by this be thought to discourage such a writer from giving his thoughts, however crude, to the public. His strength of genius, his large opportunities, and his habits of enquiry, make us anxious to see his name as often as possible. But where a work cannot be rendered complete, it would be much more desirable to meet with it in its detached state in some miscellany, which, from the manner in which it is published, admits of a ready revision from the author himself, or from the suggestions of others. If the present volume should come to a second edition, it will require some time, according to the limited circulation of medical performances. In the mean while, the author is exposed to all the obloquy of his adversaries, without the means of answering them.

---

*A brief Essay on the peculiar Advantages of the Flexible Metallic Bougies in the Treatment of Strictures in the Urethra, and the Evacuation of the Urinary Bladder. By* W. Smyth, *the Inventor,* 8vo. pp. 70. London, 1804.

Mr. Smyth gives the following account of his discovery and its progress. "Very near thirty years of extensive practice in London as an apothecary, &c. have afforded me many opportunities of witnessing the dreadful sufferings of patients labouring under obstructions of the urethra; and it was my own misfortune some time ago to be afflicted with a severe stricture, attended frequently with retention of urine, for which I could find no relief in the means recommended by my medical friends.

"It

"It is said, that 'necessity is the mother of invention;' and to this circumstance alone, perhaps, we are indebted for the discovery of my flexible metallic bougies, by the use of which I soon obtained relief, and in the course of a few weeks I was rescued from a life of misery, and restored to health and comfort.

"The gentlemen who attended me during the early part of my illness, and had left me in a very deplorable situation, on seeing me again, were much surpized at my speedy recovery, and expressed great satisfaction on viewing the instruments by which it was brought about; and judging them to be of importance in this branch of surgery, wished me to continue my endeavours to bring them to perfection.

"By unwearied perseverance this desirable object was accomplished; and as they now appeared to every examiner to be far superior to the bougies usually employed in the treatment of strictures in the urethra, some of my friends advised me to confine their use entirely to my own practice, at least until I had reimbursed myself for the expence I had been at in their construction; but I considered, that if my bougies had any real merit (which seemed to be admitted on all hands) such monopoly would not only be disagreeable to surgical practitioners in general, but might eventually be a loss to the public; I therefore sent specimens of them to the Royal College of Physicians, the Royal College of Surgeons, all the Medical Societies in London, and some surgeons of my acquaintance, for their inspection; in consequence of which I was favoured with the repeated visits of the most curious and scientific of the profession, for the purpose of investigating more minutely the advantages of a discovery, which they considered of the utmost importance to mankind.

"Honoured as I am by their patronage, no exertions on my part shall be wanting to render this pliable metal useful (as it has frequently been found) in other parts of surgery; and to have been the inventor of any thing for the improvement of the healing art, and the relief of suffering humanity, forms no small share of my present gratification."

Mr. S. after some observations on the nature and causes of stricture, proceeds to the history of bougies and the various materials of which they have been composed. He then mentions their defects and the usual objections to the incautious use of caustic, where he supports his opinions by reference and quotations from the works of most respectable surgeons. Our readers will naturally conclude, that the author's next subject will be the advantages and superiority of his own bougies, with directions for using them, and an account of the cases in which they are most successful.

In the chapter on the retention of urine, Mr. S. gives an account of the various kinds of catheters which have occasionally been used, and points out their most obvious defects. After some observations on the advantages of his own, he concludes his pamphlet with a number of testimonials from surgeons of the first respectability.

Of London Hospital Surgeons we observe the following names, which we give in the order they appear in his work, viz. Birch, Carlisle, Pearson, Howard, Blizard, Blicke, and Cline.

---

*A Complete System of Veterinary Medicine, in Two Volumes; by* James White, Veterinary Surgeon; Vol. 2. *The Materia Medica and Pharmacopœia.* 12mo. pp. 266. London, 1804.

The Title of this second volume sufficiently explains its contents, and the reputation of the author will not fail to recommend it to the attention of the public. The utility of such works, when properly executed, is sufficiently obvious, but we think our readers will not consider the following short account, given by the author himself, as superfluous.

"Within these few years only, has the Veterinary Art acquired a distinct appellation, and a solid foundation in this country. Receipts, handed down by traditionary skill, in which ingredients were accumulated without judgment or discrimination, constituted the principles and practice of what was termed farriery; a name which it derived from the occupation of the persons who practised it, who were, in general, smiths, or workers in iron *(Ferrarius.)*

"To attempt to distinguish the causes of the horse's diseases, was far beyond their little skill; and, in general, random trials of the few burning medicines in their list, formed their boasted practice.

"The science at one time began to rise above the order of smiths, and attracted the notice of medical practitioners; but it was not hereby greatly improved: they were not aware of the difference that has since been found to exist between the structure and economy of the horse and that of the human subject; nor had they any idea that this dissimilarity required much consideration with respect to disease, and the effect of medicine.* Hence they were led to bring the therapeutics and pathology of the human body to *veterinary science;* and prescribed in somewhat larger doses to the brute animal, what they had found useful to man.† Their practice was of course unsuccessful, and the art sunk into its original disrepute. It is only since the institution of the Veterinary College, that the anatomy and physiology of the horse have been properly investigated, and the effects of medicines on his body correctly

---

* One thing which never should have escaped their notice, was the horizontal position of the horse as contrasted with the upright form of man.

† Arsenic affords a striking example of this fact. In the human system, it is a deadly poison, but it may be given to the horse, even to the extent of two drachms, without danger.

White Vitriol, a strong emetic in the human body, in a small dose; may be given in the dose of eight ounces, without any violent effect. This, indeed, is the case with many other medicines, which, in man, are considered poisonous.

correctly ascertained, by numerous and appropriate experiments, both in health and disease, so that a secure foundation is now laid; and, as long as scientific men continue to study and practice the veterinary art, it must necessarily be in a progressive state of improvement.

"Notwithstanding many books have already been published concerning the diseases of the horse, the therapeutical part, or what relates to the medicines proper for his diseases, has not been hitherto explained. Such a work appeared to the author a *desideratum* in the veterinary art, and has induced him to add the present volume to his Compendium of the Diseases, &c. of which the indulgent public has already demanded a sixth edition. Having thus ventured on untrodden ground, he had no guide to lessen the labour of the attempt; but, by numerous and attentive trials, from the author's experience, and particular attention to this subject, he trusts he has been able to furnish a volume not wholly unacceptable even to the experienced practitioner. It has been the author's aim to explain the general properties of the various substances employed in medicine, accurately describing their particular effects on the body of the Horse, both in health and disease; the doses in which they may be given, their composition, and, in short, every thing that has any relation to them. This will be comprehended in the Materia Medica, or first part of the book; in the Pharmacopœia are comprized, directions for forming the various compositions in the most convenient and efficacious manner, with many valuable receipts of established efficacy under each head; the whole forming a complete system of therapeutics, instructing the inexperienced how to distinguish the purest and most genuine drugs, and to compound them in such a way, as will enable him to combat with success the various diseases to which horses are liable.

---

*An Appendix to Practical Observations on the Nature and Treatment of the exasperated Symptoms of the Venereal Disease; containing Thoughts on the Nature and Management of the Venereal Bubo, particularly in its obstinate State. By* EDWARD GEOGHEGAN, Surgeon to the Dublin General Dispensary. Octavo, pp. 44. Dublin, 1803.

IN a former Number we noticed the Treatise to which the present is an Appendix, as containing several interesting remarks on the treatment of this most important, and often most perplexing disease.

Some observations on bubo form the contents of this Appendix; and the general tendency of the author's precepts is to lessen the confidence so generally and often indiscriminately reposed on mercury as a panacea in every form of this complaint. He observes,

"As I propose to confine my observations principally to difficult cases, I shall pass over the ordinary occurrences, and suppose that matter has been formed, and its evacuation effected by a spontaneous rupture or by art; the sore, instead of discharging what is

 termed termed well conditioned matter, and having an healthy aspect, discharges an ichor, the general appearance becomes ill-conditioned, the edges ragged, and irritable, attended with great pain, and often a sloughing, and sometimes running rapidly into gangrene; this state is generally accompanied with great anxiety, restlessness, quick pulse, hot, dry skin, &c. In other instances, callous, tucked-in edges are formed, occasionally having corresponding sinusses; sometimes continuing a long time stationary, at other times spreading slowly and to great extent; exhibiting the character of herpes exedens;—under the last mentioned appearances, I have observed, that the constitution did not seem to have suffered so much as in the former; however, in all, the worst consequences are to be expected when injudiciously treated."

After quoting Hunter's practice with approbation, Mr. G. comes to the following conclusion:

"The sum of all this is, that experience has proved, that an abscess produced by the venereal poison, after its contents had been evacuated, was exasperated by the antidote to that poison, in many instances, and ultimately got well without its use; and that the same symptoms had been very generally treated by a course of mercury, and under a system of regulations, which, as to the cure of the constitutional disease, is known to be inefficacious, yet the venereal poison was removed. An instance is also given, in which a grain of mercury had not been used, and the same success attended. These circumstances, although passed over unheeded, strike me as furnishing matter, from which some useful inferences may be drawn. 'Facts and experiments, says Lord Kaims, are useless lumber, if we are not to reason about them, nor draw any consequences from them.'

"The course of reasoning, which occurs to me as arising from these observations, is, that the ulcerated buboes were not truly syphilitic, at the time they were aggravated by the antidote, and that many of these cases, which were treated in the trifling manner mentioned some years ago, were not affected at all by the mercury, but that they got well spontaneously; and the manner in which I conceive these events took place, was, that the virus was arrested in its progress by the disease it produced in the inguinal glands, and that when the contents of the bubo were evacuated in the first instance, the virus was cut off in limine, and a common abscess only remained, in which the mercury manifested deleterious instead of sanative effects; the same event I have known often to have taken place in phagædenic chancre, accompanied with phymosis; no mercury had been used, as particularly noticed in the first publication; the ulcers healed and remained well, the patients obstinately refused using mercury, which had been recommended as a security lest absorption had taken place."

The mischievous effect produced by mercury on a very irritable habit suffering under a highly painful, inflamed, irritable sore is so well understood, and so generally admitted, that this part of the author's author's reasoning, if unquestioned, is at least superfluous for any purpose of instruction. But the opinion to which he inclines, of the non-syphilitic nature of bubo in general, will bear some questioning. He observes,

"It is inculcated by every authority, and I believe it is the general practice, to commence the use of mercury immediately after the abscess has been evacuated; and notwithstanding an aggravated state of the sore should succeed, many surgeons think it advisable to persevere, until such a quantity has been used, as was usually found sufficient for the cure of the constitutional disease; bark, and the invigorating plan are generally conjoined. In healthy subjects, the aggravated state seldom occurs, the balance is mostly preserved, and the treatment seems to succeed; but when a morbid disposition previously exists, and the scale is liable to be turned on the application of any exciting cause, the injurious effects of mercury become obvious, and these instances are the tests, which develope the true character of the disease, and the just appropriation of the remedy. Mr. Hunter and others suppose a mixed state, which I think a probable circumstance; but the term is too indeterminate to afford us any satisfactory clew, it characterizes nothing, and generally encourages the exhibition of mercury. When I experience, and learn from the experience of others, that sea-bathing, country air, and the total exclusion of mercury (although little had been previously used) prove the most successful practice, and know, that a full and long continued use of it, is required to cure obstinate lues, I infer that they are not mixed cases, including the venereal poison."

We cannot but apprehend some danger from carrying this principle to its utmost extent. Though bubo may not *require* the use of mercury, the question is, whether it will *allow of* its exhibition, for the disease cannot (generally speaking) be cured without this remedy, and surely the sooner it is eradicated the better.

The author himself puts this in very strong terms, a few pages afterwards.

"With respect to the resolution of bubos by sending a current of mercury through them, every day's experience evinces, that the quantity which will suffice to remove the bubo, will not subdue the virus, but that constitutional symptoms will invariably ensue, after the idiopathic bubo has been resolved, if a sufficiency of mercury, under the necessary regulations, had not been used to have cured the constitutional disease; hence the removal of the symptoms affords no proof of the removal of the disease; and it is manifest that the virus is not destroyed in the glands. An ounce or two of ointment shall subdue the venereal irritation in the groin, and remove the bubo, and after four ounces have been used, will you not constantly find the virus in the throat or elsewhere? What's the conclusion? Surely, that it was only dislodged."

Surely then the first and principal object with the practitioner, is entirely and immediately to subdue the virus, by the only medicin medicine which can be depended on for this purpose, and not to allow slight local inconveniences to interfere with this plan, trusting to the powers of nature, to tonics, and especially to a discontinuance of the remedy, to restore the original health, when all danger from the morbid virus is done away. In no distemper is an incomplete cure more to be dreaded, since a relapse is here, not a return of the original symptoms, but a progression from bad to worse, from severe to dangerous, from troublesome to unmanageable, from the sufferings of a few weeks to the protracted misery of years, of (it may be) the remainder of life.

After all, the precise point to which mercury may and ought to be pushed, cannot be determined by any set of rules; but the propriety of the practice must arise from the circumstances of each individual case; nor will the very candid author of this little treatise venture absolutely to forbid it in all cases of suppurated bubo, but only in such as are attended with severe local inflammation. In this he would be joined by most practitioners, but not so (we apprehend) in the opinion to which he inclines, of the propriety of abstaining to give this remedy in suppurated bubo, unless other symptoms were present, characteristic of confirmed lues; since a *confirmed* lues is precisely that which the surgeon dreads, and to avoid which, all his endeavours will be most strenuously directed.

